# Examining age-related shared variance between face cognition, vision, and self-reported physical health: a test of the common cause hypothesis for social cognition

**DOI:** 10.3389/fpsyg.2015.01189

**Published:** 2015-08-12

**Authors:** Sally Olderbak, Andrea Hildebrandt, Oliver Wilhelm

**Affiliations:** ^1^Universität UlmUlm, Germany; ^2^Ernst Moritz Arndt University of GreifswaldGreifswald, Germany

**Keywords:** face perception, common cause hypothesis, fluid intelligence, immediate and delayed memory, MIMIC model, physical health, vision, face memory

## Abstract

The shared decline in cognitive abilities, sensory functions (e.g., vision and hearing), and physical health with increasing age is well documented with some research attributing this shared age-related decline to a single common cause (e.g., aging brain). We evaluate the extent to which the common cause hypothesis predicts associations between vision and physical health with social cognition abilities specifically face perception and face memory. Based on a sample of 443 adults (17–88 years old), we test a series of structural equation models, including Multiple Indicator Multiple Cause (MIMIC) models, and estimate the extent to which vision and self-reported physical health are related to face perception and face memory through a common factor, before and after controlling for their fluid cognitive component and the linear effects of age. Results suggest significant shared variance amongst these constructs, with a common factor explaining some, but not all, of the shared age-related variance. Also, we found that the relations of face perception, but not face memory, with vision and physical health could be completely explained by fluid cognition. Overall, results suggest that a single common cause explains most, but not all age-related shared variance with domain specific aging mechanisms evident.

## Introduction

The decline in fluid cognitive abilities across the adult lifespan, including particular components like mental speed, fluid intelligence, and working memory, is a well-known phenomenon (see Lövdén and Lindenberger, 2005, for a review). The decline in fluid abilities is also associated with a decrease in other cognition-related indicators such as sensory functions (i.e., vision and hearing), and physical indicators like blood pressure and respiratory functioning. While it was initially suggested that worsening cognition could be fully mediated by deteriorating sensory functions (Lindenberger and Baltes, 1994), this could not be confirmed (e.g., Anstey et al., 2001). The decline in vision does not cause the decrease in cognitive performance (Lindenberger et al., 2001), and although there is a strong relation between both functions, their age-related declines are only moderately linked (Lindenberger and Ghisletta, 2009).

An alternative explanation for the proposed downward slopes in fluid cognition, sensory functions, and physical health is that each of these factors has a unique negative relation with age. This causes each function to decline and to appear subject to a general factor, which, however, is essentially merely a statistical artifact (Salthouse et al., 1998). This explanation was supported by research that showed controlling for age reduced the relation between these variables, indicating the common cause to be more of a statistical artifact than a genuine overall factor. However, this explanation has not been supported elsewhere (e.g., Christensen et al., 2001).

As an additional perspective, many researchers propose that the decline in fluid cognitive abilities, sensory functions, and physical health indicators is indeed due to a common cause, such as the aging brain, central nervous system, or the aging body as a whole, in what is referred to as the common cause hypothesis. A strict interpretation of the common cause suggests that a single common factor explains *all* age-related shared variance between the (latent) variables of interest, particularly fluid cognitive abilities, sensory functions, and physical health indicators. Importantly, this implies that no age effects are expected for first order factors that are indicators of the common cause because age differences are completely explained by the higher order common cause factor (cf. Borsboom et al., 2003). However, tests of the common cause hypothesis do not always take this strict interpretation, with researchers finding support for the common cause hypothesis even though additional relations are needed between age and domain specific first order factors indication the postulated common cause (e.g., Christensen et al., 2001). That a common factor explains most of the age-related difference or decline in cognitive ability (e.g., fluid intelligence, working memory), sensory functions, and physical health is supported (e.g., Baltes and Lindenberger, 1997; Anstey, 1999; Anstey et al., 2001, 2003; Christensen et al., 2001; Li and Lindenberger, 2002; Valentijn et al., 2005), suggesting that the functions above are related to one another and decline with increasing age as a group. In addition, research suggests that the relations between indicators of fluid cognition, sensory functions, and physical health increase with increasing age (e.g., Baltes and Lindenberger, 1997; Li and Lindenberger, 2002).

The present paper includes an evaluation of the common cause hypothesis and competing models concerning predictions derived from this viewpoint concerning the relation between vision, physical health, and face cognition – a basic facet of social cognition. We will test two structures; the first, allowing all constructs to covary, and the second, modeling a common factor upon which all factors loads. Each structure will be tested with and without controlling for the direct effects of age on vision, physical health, and cognitive ability, and each structure will be tested controlling for fluid cognitive abilities in face cognition. All models will be presented followed by an evaluation of what the models explain regarding the relations between vision, physical health, and face cognition.

### Face Cognition

Age is associated with a stronger decline in fluid abilities (i.e., memory, attention) than crystallized abilities (i.e., basic knowledge; Baltes and Baltes, 1990; Singer et al., 2003b). Similarly, physical health has a stronger relation with fluid than crystallized abilities (Bergman and Almkvist, 2013); in a study examining the effects of a mnemonic training program on very old individuals, participants showed an improvement in fluid but not crystallized abilities (Singer et al., 2003a). The common cause hypothesis is typically evaluated with fluid abilities (i.e., working memory), but is rarely applied to more specific fluid cognitive processes. In particular, it is of interest to evaluate the extent to which the common cause hypothesis explains variance in cognitive ability factors previously shown to be distinct from traditionally established fluid cognitive abilities. As distinct ability factor, we refer to face cognition specifically, including two distinct abilities: the ability to perceive faces and the ability to remember faces (Wilhelm et al., 2010). Face cognition is considered an integral component in daily interactions and a key factor of social cognition (Beauchamp and Anderson, 2010). Face cognition has been identified as an ability that is distinct from, yet related with fluid cognitive abilities (including working memory and reasoning, object cognition, and immediate and delayed memory; e.g., Wilhelm et al., 2010). This distinction remains present across the lifespan, with relations between age and face cognition separable from the relations between age and general cognitive abilities (Hildebrandt et al., 2011). Because of their relations with general cognitive abilities, face perception, and face memory are sometimes modeled as nested factors under a general cognitive ability factor, in order to capture specific variance of face perception and face memory (e.g., Hildebrandt et al., 2011). Further, both the ability to perceive faces and the ability to remember faces can be considered fluid abilities. Face perception involves the identification of particular aspects of a face, while face memory involves perceptual processing, memory encoding and memory access and both have been modeled as indicators of a broad fluid intelligence factor (Hildebrandt et al., 2011; Kiy et al., 2013).

While we know that face cognition abilities decline with age, it is unknown how this decline relates to the decline in sensory functions or physical health. Given that both face perception and face memory are fluid abilities, we would expect the common cause hypothesis – if it holds – to also apply to these factors. That means we would expect that face cognition is related to sensory functions and physical health and ultimately to a common factor indicated by cognitive ability latent variables, sensory functions, and physical health. However, the distinction of face cognition from general cognition indicates that this may not be true, and that face cognition may have distinct relations to vision and physical health, especially after we control for the shared variance of face perception and face memory with general cognitive abilities. Thus, it is important to model face cognition in a way that controls for the general cognitive component, consequently testing the relations of age, sensory functions, and physical health with specific face perception and face memory variance only – which is new in the literature. While it is expected that face cognition will be related to sensory functions and physical health, as it has been found with general cognitive abilities and working memory, this has not been tested and as of yet is unknown.

### An Aging Brain

An aging brain is typically considered as the primary factor identified by the common cause hypothesis (e.g., Baltes and Lindenberger, 1997; Li et al., 2000) being responsible for the age-associated changes in cognition and sensory functions (e.g., Weale, 1982). The ability to perceive faces is linked with activation in the inferior occipital gyrus, the lateral fusiform gyrus, and the superior temporal sulcus (Kanwisher et al., 1997; Haxby et al., 2000; Haxby and Gobbini, 2011), each of which shows a decline in gray matter density and volume with increasing age (Raz et al., 1997; Sowell et al., 2003). Also, performance in face memory tasks, in addition to verbal memory tasks, is associated with left prefrontal cortical regions, which also are associated with a decline in volume with age (Hess, 2005).

Face perception is one of many abilities that, in younger adults, are primarily linked with activity of the ventral temporal cortex, which then shifts to the frontal regions, with reduced activation of the occipital lobe in older adults. This phenomenon is referred to as the posterior–anterior shift in aging (PASA; cf. Grady et al., 1994; Davis et al., 2008). PASA is supported by research showing that when viewing faces, houses, pseudo words, or chairs, young individuals had a high degree of neural specificity in the ventral visual cortex compared with older adults who had less neural specificity, indicating that the utilization of the fusiform gyrus for face perception was stronger for younger than older adults (Park et al., 2004). These findings were replicated by Payer et al. (2006) who also found increased activation in the middle and inferior frontal cortex in older adults.

There is ample evidence of the negative relation between age and face memory (e.g., Smith and Winograd, 1978; Grady et al., 1995; Anstey et al., 2002; de Frias et al., 2006; Hildebrandt et al., 2010); however, there is considerably less evidence regarding the relation of age with face perception. One exception is the study by Hildebrandt et al. (2011) who found that face perception, controlled for general cognitive functioning, did not show linear but instead negative quadratic age-related differences. That is, face perception abilities, when controlled for shared variance with general cognitive functioning, remained comparable between persons aged 18–60 but older persons performed worse.

### Vision

Lindenberger and Baltes (1994) found that both vision and hearing declined with increasing age and both were positively related with cognitive abilities. Vision has a stronger relation with cognition than hearing and is typically easier to measure. Common measures of vision include visual acuity, which is often measured with the Snellen test, which refers to the spatial resolution of what one can see at high contrast (i.e., the sharpness of one’s vision), and contrast sensitivity, which refers to the ability to identify certain spatial frequencies at low contrast. Researchers have found that even when assessing visual acuity and contrast sensitivity in individuals with corrected vision (i.e., individuals using glasses or contacts), both functions show an age-related decline (e.g., Owsley and Sloane, 1987).

Vision is typically considered to be associated with an age-related decline in line with the common cause hypothesis (e.g., Christensen et al., 2001). The results regarding the relation of vision with face cognition are mixed. When controlling for age, only contrast sensitivity, and not visual acuity, were identified as significant predictors in the perception of faces (Owsley and Sloane, 1987). Anstey et al. (2002) also found that when controlling for age, visual acuity was unrelated with face memory. Pfütze et al. (2002) found no relation between the speeds of face recognition with contrast sensitivity.

### Physical Health

While others typically included specific, direct measures of physical health, such as grip strength (e.g., Christensen et al., 2001), we chose a more global measure: self-reported ratings of physical health measured by the SF12. The SF12 physical health scale can reliably distinguish between clinical groups, disease severity, and (when assessed) describe recovery trajectories for individuals with rheumatoid arthritis and osteoarthritis (Hurst et al., 1998; Gandhi et al., 2001), back pain (Luo et al., 2003), retinal disease (Globe et al., 2002), HIV (Delate and Coons, 2000), acute myocardial infarction, and unstable angina (Failde et al., 2010). We chose this measure because it offers a reliable and valid assessment of general health, and instead of focusing on specific physiological measures, we have a general estimate of overall health. However, the scale is based on self-reports and hence only a proxy for physiological measures.

Self-reported health is positively related with general cognitive functioning (Zelinski and Gilewski, 2003). Individuals without mild cognitive impairment, broadly defined, report less subjective health problems, compared with those with mild cognitive impairment (Frisoni et al., 2000), and a meta-analysis of intervention programs showed that cognitive functioning in older individuals, who engaged in physical fitness activities was better than in inactive control participants (Colcombe and Kramer, 2003). The effects of health on fluid cognitive ability, however, differ in magnitude depending on the type of cognitive function assessed. For example, Bergman and Almkvist (2013) found that physical health fully mediated the effect of age on fluid intelligence, but not on crystallized intelligence. Colcombe and Kramer (2003) found that exercise had a stronger impact on performance in executive tasks, that is, tasks that require planning and inhibition, when compared with speeded and visuospatial tasks. Comijs et al. (2002) found that poor health (which they defined as the number of chronic diseases) and age predicted bad memory, even after controlling general cognitive functioning.

The relations between physical health and face cognition (including perception and learning/recognition) are not well established. One exception is a study by Bergman et al. (2007), who found a stronger relation of health with face memory than between age and face memory, with three health variables predicting 39% of the variance in face memory performance. This study suggests health is an important variable that is related with face cognition. However, this study did not control for age-related decline in general cognitive ability, thus they did not investigate specific effects of health on face memory that were not explainable through health effects on general cognitive functioning. In addition, the study was based on a relatively small sample size (*N* = 118 with persons ranging in age from 26 to 91), introducing a larger SE, and the results are not disattenuated for unreliability, suggesting the effect sizes might be higher. Nevertheless, we expected a positive relation between physical health and both face cognition factors. Furthermore, because about half of the variance in face cognition performance is explainable through general cognitive abilities (Wilhelm et al., 2010), we emphasize that the health effects on face cognition-specific variance need to be investigated after controlling for the shared variance with general cognitive abilities.

## Current Study

This paper presents a reanalysis of previously published data (see Hildebrandt et al., 2010, 2011, 2013). The relationships of face cognition abilities with health – which is the primary focus of this paper – have not been considered in any of the previous studies based on the used dataset. With structural equations modeling, we examined whether general physical health and indicators of vision are positively related with the abilities to perceive and remember faces, both before and after controlling for the effects of age and shared variance with general cognitive ability. It should be noted that, after controlling for variance due to general cognitive ability, face memory shows both a linear and a quadratic effect of age, while face perception only shows a quadratic effect of age (Hildebrandt et al., 2011). For modeling simplicity, we will include only a linear effect of age, which in the absence of the quadratic term, should capture the relative decline of face perception and face memory with increasing age.

This paper improves on the methodological shortcomings of previous studies by including multiple measures of cognitive abilities with a relatively large sample size, and the data are modeled at the level of latent variables, which are adjusted for measurement error and the specificity of the assessment method. Furthermore, we utilize structural equation modeling which allows us to model complex relations between the constructs and health-related variables.

## Materials and Methods

### Sample

Participants were 443 individuals (51% female), ranging from young (*n* = 148, ages 17–35, *M_age_* = 24.5, SD = 4.7), middle-aged (*n* = 147, ages 36–64, *M_age_* = 49.0, SD = 7.9), and older individuals (*n* = 148, ages 65–88, *M_age_* = 72.0, SD = 4.7), with the sample on average 48.5 years old (SD = 20.3). The educational background of the sample was heterogeneous, with participants who had not completed a high school degree (8%), those who have completed high school (48%), and those who have some form of college or university education (44%). All older participants performed above the cut-off score of 24 on the Mini-Mental State Examination test (Folstein et al., 1975), indicating they did not show any signs of dementia.

### Procedure and Measures

Participants completed 5 h of cognitive testing during two sessions, separated by 5–9 days. Each cognitive test came with a practice trial, during which the participants received feedback; however, no feedback was given during the actual testing trials. All tasks were administered using Inquisit 2.0^©^ with 17-inch color monitors, 85 Hz refresh rate, and 1280 × 1024 resolution. Self-report measures of health were completed at home between the two testing sessions. To this aim, participants were handed out a printed questionnaire that they were asked to complete at home and return at the second testing session. This study received approval from the Humboldt-Universität zu Berlin Psychology Department ethics committee, and written informed consent was obtained from every participant.

#### Health Measures

##### SF12 – physical health

The SF-12 is the 12-item short-form version of the SF-36 (Ware et al., 1996). Half of the SF-12 items are for the assessment of physical health status, referred to as the Physical Health scale, which is composed of four subscales: Physical Functioning, Role Physical, Bodily Pain, and General Health. An example item for the Bodily Pain subscale is “During the past week, how much did pain interfere with your normal work including both outside the home and housework?” Response options varied depending on the question (e.g., response options for the aforementioned example item were “Extremely,” “Quite a bit,” “Moderately,” “A little bit,” and “Not at all”). The internal consistency of all Physical Health items was acceptable (α = 0.831). The Physical Health scale can reliably differentiate between groups with adequate and poor physical health (Ware et al., 1996). According to the authors of the test, subscale-level scores are created by summing the items within that subscale, and one does not need to take into account any weighting scheme (Lim et al., 2008; Montazeri et al., 2009).

##### Visual acuity and contrast sensitivity

Visual acuity and contrast sensitivity were both assessed with the Freiburg Vision Test (FrACT; Bach, 2007). Both variables were assessed with the best possible optical correction, when applicable, such as the use of glasses or contacts. Visual acuity was measured according to Snellen’s fraction decimal unit and contrast sensitivity was measured by averaging the luminance of the bright and dark parts of optotypes for those trials where the participant answered correctly. Better vision is indicated by high visual acuity scores, indicating sharp vision, and higher contrast sensitivity values, indicating a better sensitivity to contrast.

#### Face Perception (FP) Tasks

All face perception and face memory tasks were developed by Herzmann et al. (2008).

##### Sequential matching of part-whole faces—conditions part (FP 1) and whole (FP 2)

In this task, participants were first presented with a target face, followed by a blank screen with an X in the middle, followed by two pictures. Those two pictures were a (1) part of the target’s face (e.g., nose) and (2) the same part from another person’s face (part condition, FP1), or they are (1) the target’s original whole face and (2) the target’s full face but with a particular part of the face (e.g., nose) replaced with that feature from another face (whole condition, FP2). The task includes 30 trials, and participants’ scores are based on how often they correctly identify the original whole face or original part of the target’s face.

##### Simultaneous matching of spatially manipulated faces—conditions upright (FP 3) and inverted (FP 4)

In this task participants were presented with a target face, followed by a blank screen with an X in the middle, followed by the same target face either in its original form or with the spatial relation between facial features (e.g., eyes and nose) altered. Half of the trials show faces upright (upright condition, FP3) and the other half presents them upside down (inverted condition, FP4). The task includes 60 trials and participants’ scores were based on how often they correctly indicated whether the two faces of a given trial were identical or not.

##### Facial resemblance (FP 5)

In this task participants were presented with a target face from a three-quarter view in the top half of the screen and two faces in the bottom left and right half of the screen. The bottom two faces are morphs of the target face, containing 20 or 40% of the target face. The task includes 48 trials and participants’ scores were based on how often they correctly identified the morphed face that contained the higher percentage of the target face.

#### Face Memory (FM) Tasks

##### Learning and immediate memory of faces (FM 1)

This task has three phases – (1) study phase (45 s), (2) unrelated task (2 min), (3) recognition phase (unlimited length) – and the sequence is presented two times, each time with new faces. During the study phase, participants were presented with 15 faces and asked to remember each one. During the subsequent recognition phase they saw each face presented during the study phase; individual faces of the memory set were presented together with a distractor face. During each of these trials participants received feedback regarding whether or not they were correct at identifying the targets. The recognition phase included five runs, each time with new distractor faces, so participants saw the target faces five times during the recognition phase (the recognition phase included 75 trials). Participants’ scores were based on how many times they correctly identified target faces during the recognition trials.

##### Delayed recognition of learned faces 1 and 2 (FM 2 and FM 3)

This task is a continuation of the *Learning and immediate memory of faces* (FM1) task. Participants repeated the recognition phase of FM1, with new distractor faces. This was done at the end of the first test session, about 2.5 h after the initial learning phase (FM2) and at the beginning of the second test session (FM3). Both FM2 and FM3 have 30 trials each and scores were based on how often participants correctly identified the target face.

##### Eyewitness testimony (FM 4)

In this task participants were presented with two faces, one of which was seen during an earlier face cognition speed task. This task consists of 46 trials and participants’ scores were based on how often they correctly identified which face they had seen before.

#### Working Memory (WM) and Reasoning (REA) Tasks

##### Memory Updating (WM 1)

In this task, adapted from Oberauer et al. (2000), participants were presented with a 3 × 3 grid with single-digit numbers presented consecutively in each cell. Participants were asked to memorize those numbers and then, in a series of visual instructions, arrows pointing upward or downward appeared in each cell requiring participants to mentally update the numbers in the cells by either adding or subtracting 1, respectively. At the end of these instructions, participants were asked to type in the new numbers into each cell. This process was done 18 times and participants’ scores were based on how many correct responses they had provided.

##### Rotation Span (WM 2)

In this task, adapted from Kane et al. (2004), participants were presented with a sequence of arrows that they were to memorize (specifically the arrows’ length and direction), while simultaneously completing a secondary task where they decided whether a letter was presented in mirror form or not. Participants’ scores were based on the proportion of correctly recalled arrow positions and lengths.

##### Raven’s Advanced Progressive Matrices (REA 1)

Sixteen trials, from Raven et al. (1979), were presented (five trials differed between the participants, with older adults receiving easier trials compared to young and middle-aged participants). In each trial, a 3 × 3 matrix was presented, including symbols with one symbol in the bottom right missing. From eight options participants selected, which symbol logically completed the matrix. One-third of the items differed between the participants depending on their age. Older participants completed only 10 of the 15 difficult items, compared with the younger and middle aged adults who completed all 15 difficult items. Because the older participants worked on different items, the participants’ scores were based on a linked 2-Parameter Logistic Model.

#### Immediate and Delayed Memory (IDM) Tasks

The following three tasks are based on the Wechsler Memory Scale (Härting et al., 2000).

##### Verbal memory – immediate (IDM 1) and delayed (IDM 2)

This task consisted of three sequences of a learning and recall phase, containing the same eight words pairs each time, but ordered differently. During the learning phase, participants heard the eight word pairs that they should memorize (we used eight instead of the original six trials to avoid ceiling effects in the younger sample). During the recall phase, immediately following each learning phase, participants heard one word from the word pair and were asked to type the second word of the word pair (condition immediate, IDM1). About 1.5 h later, participants were again asked to complete the recall phase (condition delayed, IDM2). Participants’ scores were based on how often they typed in the correct word pair.

##### Name memory – immediate (IDM 3) and delayed (IDM 4)

This task has the same structure as the verbal memory task. However, instead of pairs of words, participants are to memorize written first and last name combinations and to recall the last name when the first name was presented in written form. Participants were asked to either immediately recall the last name (condition immediate, IDM3) and 1.5 h later (condition delayed, IDM4). Scores were based on how often participants typed in the correct word pair.

##### Address memory – immediate (IDM 5) and delayed (IDM 6)

The structure of this task is the same as that of the name memory task, but instead of pairs of names, participants read and memorized street names and corresponding house numbers and should recall the house numbers when the street name was presented on the screen. Participants were recall the house numbers either immediately (condition immediate, IDM5) and again 1.5 h later (condition delayed, IDM6). Participants’ scores were based on how often they typed in the correct house number.

## Results

### Missing Data

All data were visually screened for outliers in univariate and bivariate distributions and outliers were set to missing. Specifically, values more than 3.5 standard deviations from the mean and scale-level performance scores that were below guessing probability were set to missing. All missing data was imputed with the Expectation–Maximization algorithm available in SPSS. The accuracy tasks were missing 45 values out of 8064 (1% of the observations; see Hildebrandt et al., 2011 for details). The SF12 was missing 75 values out of 5316 (1% of the observations). Visual acuity and contrast sensitivity were missing for 20 persons (5% of the sample), thus 40 data points out of 886. Please see Appendix **Table A1** for the final covariance matrix.

### Measurement Models

All analyses were performed within Mplus Version 7 and model fit was evaluated based on standards suggested by Hu and Bentler (1999) with SRMR ≤ 0.08, RMSEA ≤ 0.06, CFI ≥ 0.95, and TLI ≥ 0.95 indicating the model is a good fit to the data and SRMR ≤ 0.10, RMSEA ≤ 0.08, CFI ≥ 0.90, and TLI ≥ 0.90 indicating the model is an acceptable fit to the data. The chi-square was evaluated according to the ratio of the chi-square value to the degrees of freedom, with ratios 2.5:1 or lower indicating acceptable fit. In order to investigate the effects of age on cognition we employed two measurement model structures. The first tested simple measurement models, with each construct indicated by its specific cognitive tasks. The second employed a nested model structure with a single factor representing general cognitive ability and nested face perception, face memory, and immediate and delayed memory. In this structure, the general cognitive ability factor was indicated by the working memory and reasoning tasks, with each indicator of face perception, face memory, and immediate and delayed memory also loading on the general cognitive ability factor. That general cognitive ability is based on these measures indicates that the factor can be considered a measure of fluid cognition. In both types of measurement models, we allowed the residuals between tests (i.e., the indicators) that shared similar assessment characteristics to covary: the upright and inverted conditions of the simultaneous matching of spatially manipulated faces task, and the verbal and learning recognition portions within each of the immediate and delayed memory tasks.

The measurement model for face perception fit the data well [χ^2^_(4)_ = 4.63, *p* = 0.33, SRMR = 0.015, RMSEA = 0.019, CFI = 0.998, TLI = 0.996, AIC = -3835.95, BIC = -3770.45], with loadings moderate to strong (λs ranged from 0.474 to 0.664). The measurement model for face memory also fit the data well [χ^2^_(2)_ = 3.52, *p* = 0.17, SRMR = 0.008, RMSEA = 0.041, CFI = 0.999, TLI = 0.996, AIC = -3908.31, BIC = -3859.19], with all loadings strong (λs ranged from 0.690 to 0.918). And the measurement model for immediate and delayed memory fit the data well [χ^2^_(6)_ = 12.33, *p* = 0.06, SRMR = 0.010, RMSEA = 0.049, CFI = 0.996, TLI = 0.991, AIC = -2279.86, BIC = -2193.90], with all loadings strong (λs ranged from 0.624 to 0.865). The fluid cognitive ability measurement model was indicated by only three indicators, thus exhausting all degrees of freedom so its fit cannot be tested in a separate measurement model; loadings were strong (λs ranged from 0.758 to 0.824). Finally, the measurement model with all cognitive ability factors modeled simultaneously in a nested structure had acceptable fit to the data [χ^2^_(116)_ = 313.74, *p* < 0.05, SRMR = 0.062, RMSEA = 0.062, CFI = 0.958, TLI = 0.945, AIC = -7441.42, BIC = -7142.59], with general cognitive ability factor loadings moderate to strong (λs ranged from 0.317 to 0.790), weak to strong for the nested face perception factor (λs ranged from 0.170 to 0.538), moderate to strong for the nested face memory factor (λs ranged from 0.497 to 0.690) and moderate to strong for the nested immediate and delayed memory factor (λs ranged from 0.342 to 0.585).

Vision was modeled as a single latent variable indicated by visual acuity and contrast sensitivity. Because the construct was indicated by only two indicators, both variables were standardized and the loadings were equated (both loadings were 0.811). Again, this model is just identified.

Finally, self-reported physical health was modeled based on the latent factor structure by Montazeri et al. (2009). Here, one latent variable, representing physical health, was indicated by all four subscales. The model fit was comparable to that described by Montazeri et al. (2009): χ^2^_(2)_ = 7.95, *p* < 0.05, RMSEA = 0.082, CFI = 0.991, TLI = 0.972, with all loadings being moderate to strong in magnitude (λs ranged from 0.573 to 0.828). The RMSEA is considered poor fit by Hu and Bentler (1999), mediocre by MacCallum et al. (1996), and good fit by Steiger (1989). The RMSEA can be inflated when a model has incorrectly omitted a single covariance between residuals (Savalei, 2012). An examination of the modification indices suggested that the addition of a covariance between the residuals of Role Physical and Bodily Pain, which was weak in magnitude (*r* = 0.278, *p* < 0.05), resulted in a lower RMSEA value [χ^2^_(1)_ = 0.22, *p* = 0.64, SRMR = 0.003, RMSEA = 0.000, CFI = 1.000, TLI = 1.007; note: the TLI can fall out of the range of 0–1; Kline, 2005]. However, we decided against including this covariance because it was not specified a priori (Steiger, 1990) or postulated in the model by Montazeri et al. (2009), and excluding this covariance should not impact the basic correlational pattern between latent factors (Newcomb and Bentler, 1988).

### Measurement Invariance

We employed restricted factor models (RFMs), a special condition of latent moderated structures analysis (Klein and Moosbrugger, 2000), where we included a measured variable as a moderator of measurement and structural model parameters to test measurement invariance across age. RFM works by allowing the creation of an interaction variable between age and the particular moderator of interest (e.g., self-reported physical health) and regressing the variables predicted by age and suspected of having an age effect moderated into the interaction term. RFM allows us to test the effect of moderators in a latent variable context while keeping the components of the interaction variable continuous. RFM analyses, however, do not come with established fit indices (e.g., RMSEA; instead, they come with -2 Log Likelihood, AIC, and BIC) so usually the models are first computed with just the main effects, and no interaction variables, with Maximum Likelihood estimation to establish the fit of the model. Then, the modeled is re-estimated within the RFM framework, to provide a baseline of model fit without the interaction variables, followed by a third model estimated within the RFM framework that includes the interaction variables. Nested models in RFM are compared based on likelihood ratio tests. It should be noted that RFM analyses cannot provide standardized estimates of path coefficients; instead, we present *t* values.

The measurement invariance of face perception, face memory, immediate and delayed memory, and general cognitive ability across age were previously established and the results are presented in Hildebrandt et al. (2011). The measurement invariance of physical health across age was estimated with RFM analyses. First, the measurement model of physical health was re-estimated within the RFM framework with age as a direct predictor of physical health (model PH1; see **Table 1** for model fit). Then, an interaction term between physical health and age centered was created, and all indicators of physical health were regressed on this interaction (model PH2). Three of the four loadings onto the interaction term were statistically significant indicating that the loading of that indicator on physical health changes due to age (see **Table 2**). Thus, the subscales Physical Functioning, Role Physical, and Bodily Pain increase in relation to the latent factor physical health, with increasing age.

**Table 1:** Model fit from restricted factor model (RFM) measurement invariance analyses.

Model	L	SCF	FP	Δχ2	Δdf	AIC	BIC
PH1 – Physical health with age	-3243.46	1.34	15	–	–	6516.92	6578.49
PH2 – Age moderates the loadings of physical health indicators on physical health	-3191.48	1.66	19	36.39^∗^	4	6420.97	6498.96

*^∗^*p* < 0.05; L, Loglikelihood value; SCF, scaling correction factor; FP, number of free parameters; AIC, Akaike’s information criterion; BIC, Bayesian information criterion.*

**Table 2:** Effect sizes (expressed as *t* values) of the interacting effect of age on the loadings of the physical health indicators on the latent variable physical health.

Physical health (Model PH2)				
	Physical functioning	Role physical	Bodily pain	General health
Physical health ^∗^Age	**9.616^∗^**	**3.614^∗^**	**3.082^∗^**	0.456

*Bolding indicates statistical significance (^∗^*p* < 0.05).*

To test whether the inclusion of this interaction term significantly improved fit, we estimated Δχ^2^ values from the earlier model (PH1) where the interaction term was not included. Because both models were estimated within the RFM framework, we used the following formula (e.g., Muthén and Muthén, 2015):

$$\Delta \chi^{2}=\frac{2^{*}(L_{\mathrm{modelB}}-L_{\mathrm{modelA}})}{\left({\mathrm{SCF}}_{\mathrm{modelA}}{}^{*}{\mathrm{FP}}_{\mathrm{modelA}})-({\mathrm{SCF}}_{\mathrm{modelB}}{}^{*}{\mathrm{FP}}_{\mathrm{modelB}})/(FP_{\mathrm{modelA}}-{\mathrm{FP}}_{\mathrm{modelB}}\right)}$$

where L is the log-likelihood, SCF is the scaling correction factor, and FP is the number of free parameters. The Δχ^2^ between the two models was statistically significant, indicating that the inclusion of the moderation effects of age significantly improved model fit. Three of the four loadings were statistically significant, indicating that the relations of those indicators with the central construct of physical health, and essentially with each other, increases with age. This suggests that metric invariance is partly supported.

### Structural Models

#### Models 1A and 1B – Covariances between Cognitive Ability (Including Face Perception and Memory), Vision, and Physical Health

**FIGURE 1:**
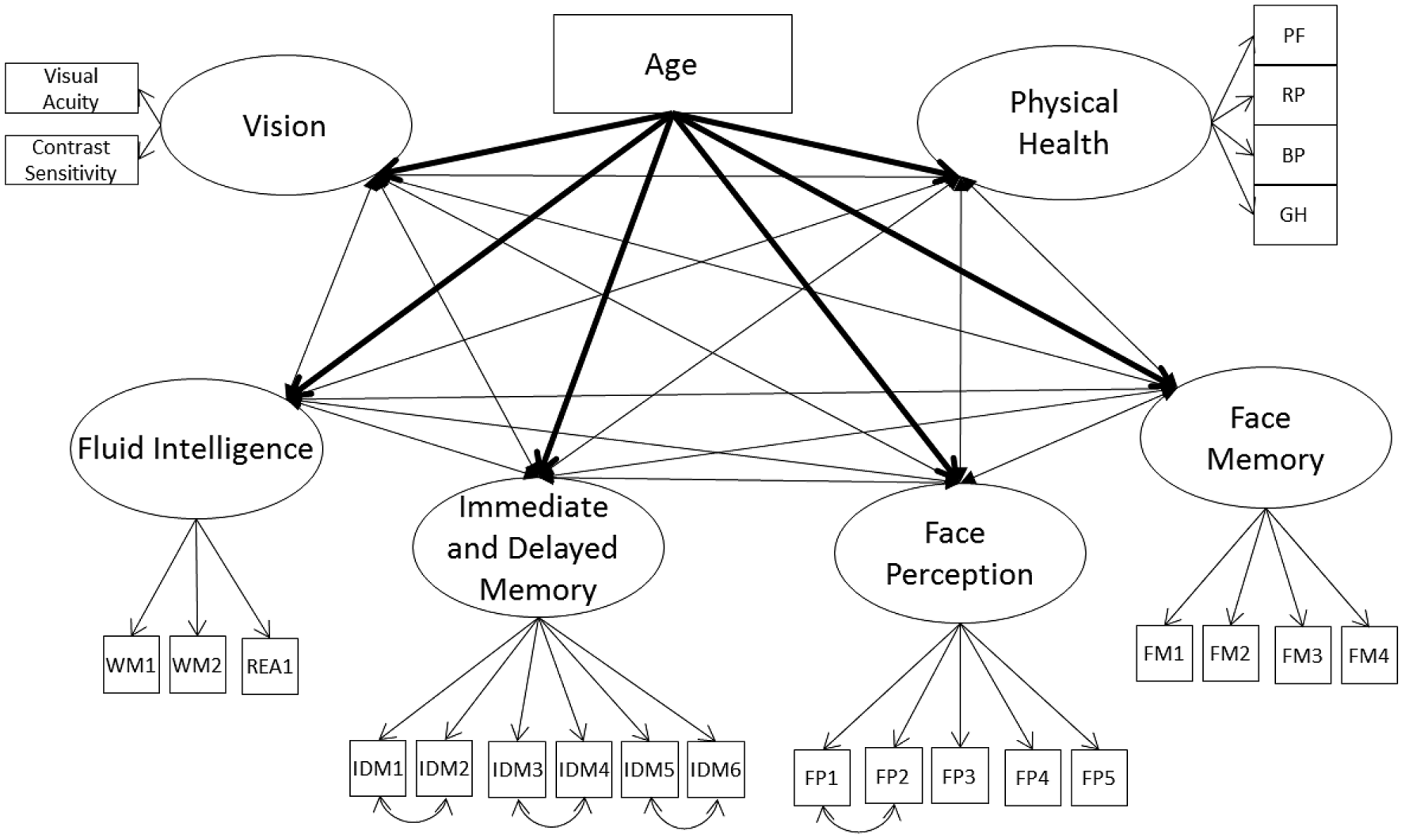
**Schematic representation of Models 1A and 1B (residual variances are not displayed), with the cognition factors, vision, and physical health covarying and in Model 1B only a direct effect of on all factors (indicated with bolded lines)**.

First, we tested whether there was convergence between the cognitive ability factors, modeled with their individual non-nested measurement model, vision, and physical health (Model 1A; see **Figure 1**). The model fit the data well (see **Table 3**), with all cognitive ability factors strongly related to one another and with vision. Also, the cognitive ability factors and vision were weakly to moderately related with physical health (**Table 4**). Next, we tested whether these relations decrease once we control for the effects of age. We included age as a direct predictor of each factor and correlated the factor residuals (Model 1B; see **Figure 1** and **Table 4**). The model fit the data well (see **Table 3**) and age had a moderate to strong effect on all of the factors. Overall, the relations between the constructs were reduced, and particularly for self-reported physical health, eliminated completely. Fluid intelligence, immediate and delayed memory, and face perception were moderately related with one another, while face memory was now weakly related with fluid intelligence, moderately related with immediate and delayed memory, and strongly related with face perception. The relations of cognitive ability with vision dropped from strong effect sizes to weak, with immediate and delayed memory no longer significantly related with vision. Finally, the only remaining significant relation with self-reported physical health was for fluid intelligence. Overall, these results suggest that there might be a common factor that includes face perception and face memory in addition to fluid intelligence, immediate and delayed memory, vision, and physical health. That factor should remain influential even after controlling for the effects of age, however, it would be expected that when controlling for the effects of age that physical health is no longer related to the common factor.

**Table 3:** Model fit.

Model	χ^2^	df	SRMR	RMSEA	CFI	TLI	AIC	BIC
(1A) All cognition factors, vision, and physical health covary	382.64^∗^	234	0.045	0.038	0.975	0.971	-2947.69	-2579.27
(1B) All cognition factors, vision, and physical health are predicted by age and covary	459.62^∗^	252	0.046	0.043	0.968	0.962	538.20	939.37
(2A) Immediate and delayed memory, face perception, and face memory are nested under the general cognition factor, and immediate and delayed memory, face perception, face memory, and general cognition covary with vision and physical health, and vision and physical health covary	497.37^∗^	225	0.060	0.052	0.954	0.944	-2814.96	-2409.70
(2B) Immediate and delayed memory, face perception, and face memory are nested under the general cognition factor, all cognition factors, vision, and physical health are predicted by age, and immediate and delayed memory, face perception, face memory, and general cognition covary with vision and physical health, and vision and physical health covary	570.48^∗^	243	0.062	0.055	0.949	0.938	-3260.70	-2830.86
(3A) All cognition factors, vision, and physical health load on a common factor	454.77^∗^	243	0.052	0.044	0.965	0.960	-2893.57	-2561.99
(3B) All cognition factors, vision, and physical health load on a common factor and the common factor, as well as fluid intelligence, immediate and delayed memory face memory, vision, and physical health, are predicted by age	517.96^∗^	261	0.052	0.047	0.960	0.954	578.54	942.86
(4A) Immediate and delayed memory, face perception, and face memory are nested under the general cognition factor, with vision, physical health, and general cognition loading on the common factor	522.56^∗^	231	0.059	0.053	0.951	0.942	-2801.77	-2421.07
(4B) Immediate and delayed memory, face perception, and face memory are nested under the general cognition factor, with vision, physical health, general cognition, and nested face memory loading on the common factor	501.58^∗^	230	0.061	0.052	0.955	0.945	-2820.75	-2435.96
(4C) Immediate and delayed memory, face perception, and face memory are nested under the general cognition factor, with vision, physical health, general cognition, and nested face memory loading on the common factor with age predicting the common factor and nested immediate and delayed memory	549.36^∗^	250	0.064	0.052	0.954	0.945	631.93	1041.29

*^∗^*p* < 0.05.*

**Table 4:** Correlations between cognition, vision, and physical health before (Model 1A) and after (Model 1B) controlling for age, expressed as fully standardized β values.

	Fluid intelligence	Immediate and delayed memory	Face perception	Face memory	Vision	Self-reported physical health
**Covariates**						
Immediate and delayed memory	**0.729^∗^/0.491^∗^**					
Face perception	**0.699^∗^/0.466^∗^**	**0.643^∗^/0.408^∗^**				
Face memory	**0.549^∗^/0.163^∗^**	**0.659^∗^/0.416^∗^**	**0.829^∗^/0.718^∗^**			
Vision	**0.602^∗^/0.154^∗^**	**0.540^∗^/**0.128	**0.600^∗^/0.282^∗^**	**0.610^∗^/0.271^∗^**		
Self-reported physical health	**0.328^∗^/0.119+**	**0.217^∗^/**-0.007	**0.265^∗^/**0.071	**0.263^∗^/**0.058	**0.273^∗^/**0.033	

Direct effect of age (for Model 1B only)	**-0.727^∗^**	**-0.646^∗^**	**-0.613^∗^**	**-0.640^∗^**	**-0.728^∗^**	**-0.350^∗^**

Values left of the / indicate coefficients from Model 1A, and values on the right of the / indicate coefficients from Model 1B, with the exception of the Age row, which only includes coefficients from Model 1B. Bolding indicates statistical significance (*p < 0.05; +*p* = 0.05).

#### Models 2A and 2B – Covariances between Cognitive Ability (Modeled with Nesting), Vision, and Physical Health

**FIGURE 2:**
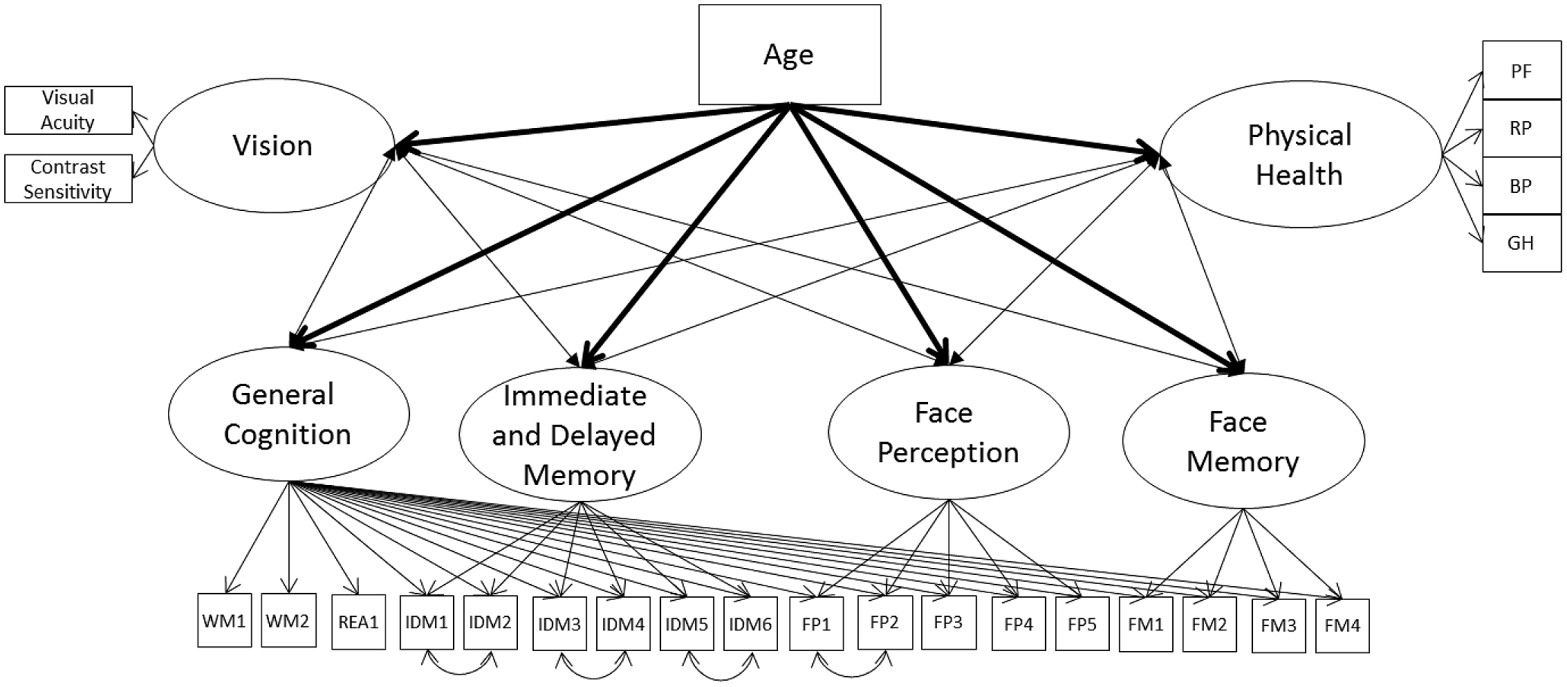
**Schematic representation of Models 2A and 2B (residual variances are not displayed), with the cognition factors, vision, and physical health covarying and in Model 2B only a direct effect of on all factors (indicated with bolded lines)**.

Face perception and face memory might be related with vision because they are fluid abilities and not because of their content-related specificity; therefore, we next remodeled Model 1A with the nested cognitive ability factor instead of the separate measurement models for cognitive abilities (Model 2A, see **Figure 2**). The model had acceptable fit to the data (**Table 3**) with the general cognition factor significantly related to vision and physical health. The nested cognitive ability factors were not significantly related to vision or physical health with the exception of face memory, which was significantly related with vision, suggesting that the significant relations between face cognition and vision were mainly due to their general fluid ability component (**Table 5**). Next, we remodeled Model 2A with age as a direct predictor of all latent factors. This model had acceptable fit to the data (**Table 3**). Age was a significant predictor of all latent variables, with the exception of the nested face perception factor (**Table 5**). Controlling for the direct linear effects of age reduced the magnitude of almost all correlations. The general cognition factor was still significantly related with physical health and vision, and the nested face memory factor was still significantly related with vision, but all correlations were now weak in magnitude. Also, the relation between vision and physical health was no longer statistically significant.

**Table 5:** Correlations between cognition, vision, and physical health before (Model 2A) and after (Model 2B) controlling for age, expressed as fully standardized β values.

	General cognition	Immediate and delayed memory	Face perception	Face memory	Self-reported physical health	Vision
**Covariates**						
Self-reported physical health	**0.343^∗^/0.126^∗^**	-0.103/-0.082	-0.083/-0.046	0.087/0.050		
Vision	**0.651^∗^/0.199^∗^**	-0.017/-0.052	-0.019/0.068	**0.241^∗^/0.207^∗^**	**0.282^∗^/**0.039	

Direct effect of age (for Model 2B only)	**-0.732^∗^**	**-0.329^∗^**	-0.177	**-0.461^∗^**	**-0.349^∗^**	**-0.729^∗^**

Values left of the / indicate coefficients from Model 2A, and values on the right of the / indicate coefficients from Model 2B, with the exception of the Age row, which only includes coefficients from Model 2B. Bolding indicates statistical significance (*p < 0.05).

#### Models 3A and 3B – Common Factor with Face Perception and Face Memory

**FIGURE 3:**
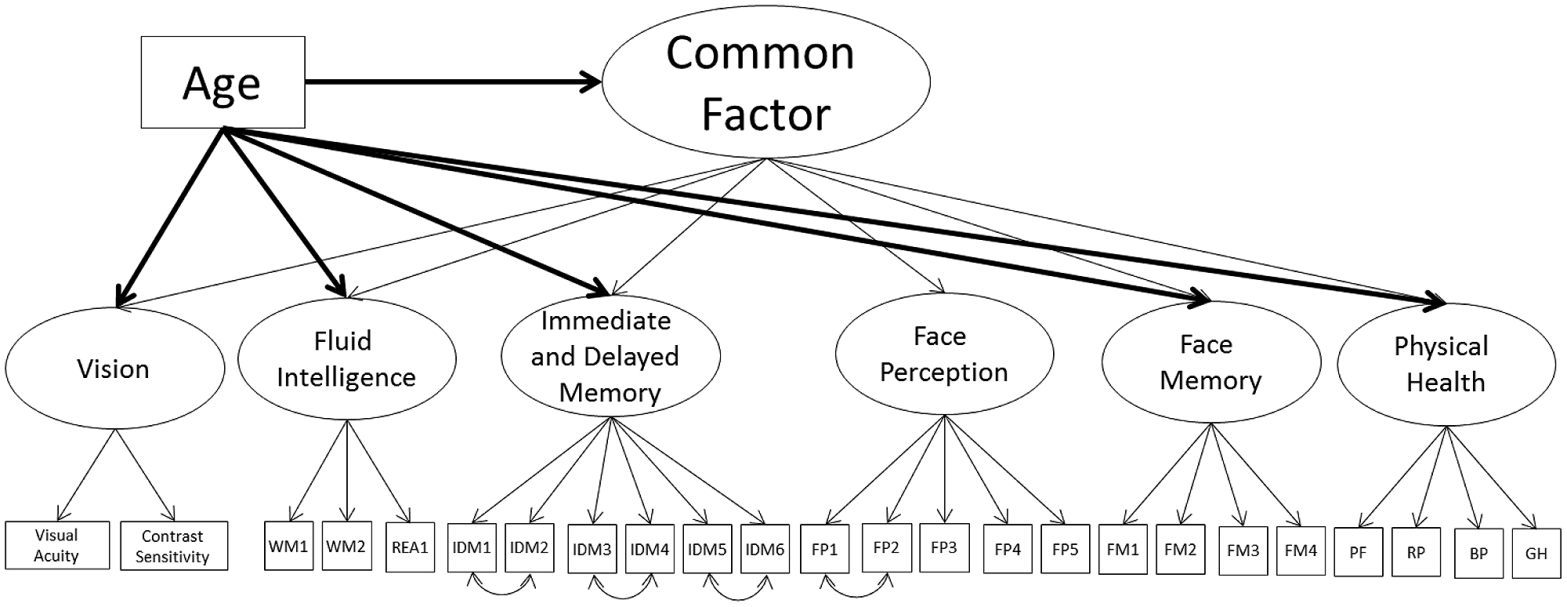
**Schematic representation of Models 3A and 3B (residual variances are not displayed), with a single second-order factor, labeled the common factor, predicting cognition, vision, and physical health and in Model 3B only a direct effect of age all factors (indicated with bolded lines)**.

Given Models 1A, 1B, 2A, and 2B still present significant correlations, even after controlling for age, we next modeled a second-order common factor structure. In the first model, the common factor was indicated by face perception, face memory, fluid intelligence, immediate and delayed memory, vision, and physical health (Model 3A; see **Figure 3**; **Table 6**). The model fit the data well (**Table 3**). All cognitive ability factors and vision were strongly related to the common factor, with self-reported physical health moderately related, and all factors had a significant proportion of variance accounted for by the common factor: 62% of fluid intelligence, 63% of immediate and delayed memory, 81% of face perception, 68% of face memory, 51% of vision, and 11% of physical health.

**Table 6:** Common factor loadings before (Model 3A) and after (Model 3B) controlling for age, expressed as fully standardized β values.

	Vision	Fluid intelligence	Immediate and delayed memory	Face perception	Face memory	Self-reported physical health	Common factor
Common factor loadings	**0.715^∗^/0.284^∗^**	**0.785^∗^/0.394^∗^**	**0.795^∗^/0.522^∗^**	**0.902^∗^/0.965^∗^**	**0.825^∗^/0.721^∗^**	**0.328^∗^/**0.098	
Direct effect of age (for Model 2B only)	**-0.548^∗^**	**-0.478^∗^**	**-0.315^∗^**		**-0.183^∗^**	**-0.288^∗^**	**-0.634^∗^**

Values left of the / indicate coefficients from Model 3A, and values on the right of the / indicate coefficients from Model 3B, with the exception of the Age row, which only includes coefficients from Model 3B. Bolding indicates statistical significance (*p < 0.05).

Next, we employed a Multiple Indicator Multiple Cause (MIMIC; Muthén, 1988) model to model the effects of age on the common factor and each of the common factor indicators, to see if the relations of any of the indicators with the common factor are reduced or eliminated with the inclusion of age (Model 3B; see **Figure 3**; **Table 6**). To identify the model we constrained the relation of age on face perception to zero, thus all additional effects of age on the common factor indicators can be compared relative to face perception. In other words, the effect of age on face perception is modeled through the relation of age on the common factor, and the additional effects of age on the common factor indicators (i.e., face memory) are in addition to the effect of age on the common factor. Recommendations suggest choosing a reference factor that is not expected to have an additional effect of age outside of the relation mediated by the common factor. The direct effects of age in Model 1B would suggest using self-reported physical health as the reference variable, because it showed the weakest effect of age. However, the inclusion of age will most likely lead to self-reported physical health not significantly related to the common factor. Therefore, we instead chose the performance indicator with the lowest effect of age, face perception, which also maintained its relation with the common factor even after the direct effects of age were modeled.

The model fit the data well (see **Table 3**). There was a strong negative effect of age on the common factor, with additional negative relations for face memory, fluid intelligence, immediate and delayed memory, and vision. These additional effects of age are similar to the pattern of age effects in Model 1B. There was a weak additional effect of age on physical health, however, like in Model 1B, self-reported physical health was now not significantly related to the common factor; hence this relation is not in addition to the effect of age on the common factor. Most importantly, face perception and face memory were still strongly related to the common factor, with fluid intelligence moderately related, immediate and delayed memory strongly related, and vision weakly related, however, the proportion of variance in all factors explained by the common factor was now reduced. The proportions were 16% of fluid intelligence, 27% of immediate and delayed memory, 93% of face perception, 52% of face memory, 1% of physical health, and 8% of vision. However, despite the reduced relations with the common factor, these results suggest that face cognition is part of the common factor, even after controlling for the effects of age.

#### Models 4A, 4B, and 4C – Common Factor with Nested Face Perception and Face Memory Factors

**FIGURE 4:**
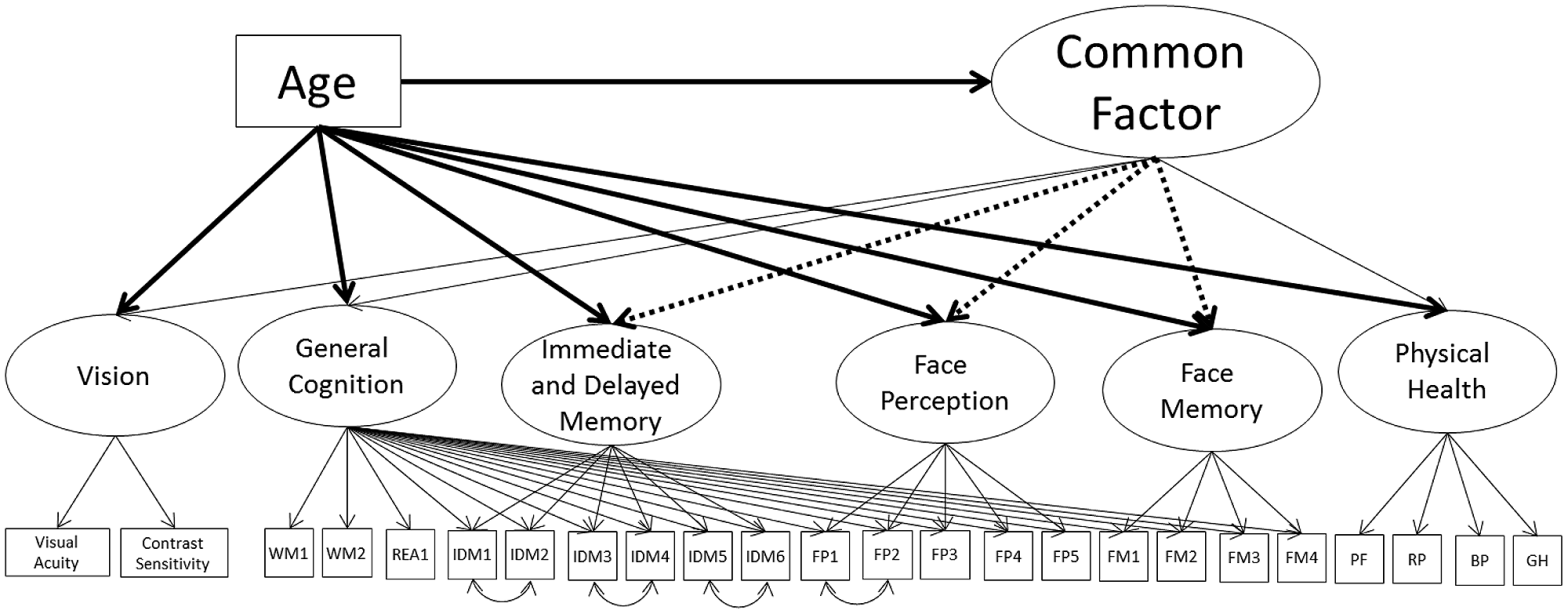
**Schematic representation of Models 4A, 4B, and 4C (residual variances are not displayed).** Each model postulates a single second-order factor, labeled the common factor, predicting vision, general cognition, and physical health. The dotted lines shows the additional paths tested for Model 4B. The bolded paths show the effects of age tested for Model 4C.

Next, we remodeled the common factor but with the nested cognitive ability factor instead of the separate cognitive ability measurement models (Model 4A; see **Figure 4**; **Table 7**). The model had acceptable fit to the data (see **Table 3**). Vision and general cognitive ability were strongly related to the common factor, self-reported physical health moderately related, and the common factor explained 55, 84, and 13% of the variance in the factors respectively.

**Table 7:** Common factor loadings with a nesting structure, before controlling for age (Models 4A and 4B) and after (Model 4C), expressed as fully standardized β values.

	Vision	General cognition	Immediate and delayed memory	Face perception	Face memory	Self-reported physical health	Common factor
Common factor loadings	**0.744^∗^/0.760^∗^/0.766^∗^**	**0.915^∗^ /0.862^∗^/0.781^∗^**			NA**/0.810^∗^/0.791^∗^**	**0.367^∗^/0.359^∗^/0.348^∗^**	
Direct effect of age (for Model 3C only)			**-0.490^∗^**				**-0.889^∗^**

Values left of both / indicate coefficients from Model 4A, and values in between the / indicating coefficients from Model 4B, and values on the right of both / indicating coefficients from Model 4C, with the exception of the Age row, which only includes coefficients from Model 4C. NA indicates this value was not estimated. Bolding indicates statistical significance (*p < 0.05).

Next, we tested the inclusion of paths from the common factor to the nested face perception, face memory, and immediate and delayed memory factors. To keep the structure stable, paths were tested one at a time. Only the nested face memory factor was significantly related to the common factor, with a strong relation to the common factor (66% of the variance explained), with this model having acceptable fit to the data (Model 4B; see **Table 7**). This model suggests that the relation of face perception and immediate and delayed memory with the common factor is fully mediated by the general fluid cognitive ability factor, with the nested face memory factor also having a direct relation with the common factor.

To see if any of these relations change with the inclusion of age, we again modeled age as a direct predictor of the common factor, and iteratively included direct relations of age on each of the cognitive ability factors (Model 4C; see **Table 7**). Like with Model 3B, there was a strong negative effect of age on the common factor, with an additional moderate negative effect on the nested immediate and delayed memory factor (model fit was acceptable). There were no additional effects of age on the nested face perception or face memory factors, and the magnitudes of the loadings on the common factor were similar in magnitude to Model 4B. These results suggest that the effects of age on face perception can be fully mediated by the effect of age on the common factor and the strong relation of the general cognitive ability factor (under which face perception is nested) with the common factor. That there are no additional significant effects of age on face memory indicates that when modeled as a nested factor, the relation of face memory with age is fully mediated by the common factor. The additional effect of age on immediate and delayed memory suggests that that the effect of age on immediate and delayed memory cannot be fully explained by the common factor or by the general cognitive ability component of this factor.

## Discussion

### Summary

We presented a succession of models testing the relation between face perception, face memory, fluid cognitive ability, and immediate and delayed memory, vision, and physical health with each other and with age. These models differed in model fit, with no agreement amongst an evaluation of the fit indices in terms of the best fitting model. According to the AIC and BIC, Model 2B was the best fit to the data, however, according to SRMR, RMSEA, CFI, and TLI, Model 1B was the best fit. However, all models had acceptable fit to the data, according to the SRMR, RMSEA, CFI, and TLI, suggesting that while the structures differ, each offered an acceptable description of the data. Models 1A, 1B, 2A, and 2B, which presented strictly correlational structures, found significant correlations between the latent constructs, which is indicative of shared variance, although that shared variance was not directly modeled. Models 3A, 3B, 4A, 4B, and 4C presented a hierarchical latent factor structure, which did model the shared variance amongst the latent variables labeling this shared variance as a common factor. While the structures differ, they are merely different approaches to modeling the variance shared amongst the latent factors, specifically cognitive ability, vision, and physical health. Next, we will present an evaluation of what these models indicate regarding the relations between fluid abilities, in particular face cognition, with vision and health.

#### Effects of Age

Model 1B and 2B indicated a strong negative linear trend of age on vision supporting existing findings (e.g., Owsley and Sloane, 1987; Lindenberger and Baltes, 1994) that vision decreases with age. Model 3B indicated that when vision was modeled as part of a common factor, which was also indicated by physical health and all of the cognitive ability factors (modeled in a non-nested structure), age had an effect on vision in addition to the effect mediated through the common factor. The final model (Model 4C), however, illustrated that when the common factor is indicated by a stronger fluid cognitive ability factor (labeled general fluid cognitive ability), a nested face memory factor, physical health, and vision, that age no longer had an additional effect on vision and instead the common factor fully mediated the effect of age on vision. That Model 3B showed a unique direct effect of age on vision, in addition to the effect mediated through the common factor, supports the findings of Christensen et al. (2001) and the results of Model 4C suggest that if Christensen and colleagues had remodeled their common factor with a stronger fluid cognitive ability factor, they might not have found a direct effect of age on vision.

For physical health, Model 1B and 2B indicated a moderate negative linear trend of age, supporting existing findings that general health declines with age. Model 3B indicated that when the common factor is indicated by vision and all of the cognitive ability factors (modeled in a non-nested structure), age was a stronger predictor of the common factor than physical health. The final model (Model 4C), however, indicated that when the common factor was indicated by a stronger fluid cognitive ability factor, a nested face memory factor, physical health, and vision, that age was no longer a stronger predictor of physical health when compared with the common factor. Instead, the effects of age on physical health could be fully mediated by the common factor.

Model 1B and 2B illustrated a strong negative effect of age on fluid cognitive ability, immediate and delayed memory, face perception, and face memory, supporting research that fluid cognitive abilities decline with age. In Model 3B, in addition to the strong negative effect of age on the common factor, which was moderately to strongly indicated by each cognition factor, all of the cognitive ability factors (with the exception of face perception which will be discussed in more detail below) had additional effects of age that were not mediated by the common factor. In Model 4C, however, when we employed a nesting structure for the cognitive ability factors, the effects of age on general cognitive ability, face perception, and face memory were fully mediated by the common factor, with only immediate and delayed memory showing an additional effect of age.

The lack of an additional effect of age on face perception in Model 3B highlights the findings by Hildebrandt et al. (2011) that face perception (controlled for general fluid cognitive ability) only shows a significant quadratic trend of age and not a significant linear trend. While face perception did show a strong negative effect of age in Model 1B, it is important to note that in that model, face perception ability was estimated without the use of a nested structure, thus without controlling for age effects on general cognitive functioning. Models 2B and 4C employed a nesting structure and found no linear effect of age on face perception. Likewise, Model 3B, through the common factor that was indicated by fluid cognitive ability and other fluid abilities, essentially partialled out the fluid ability aspects of face perception, and the remaining face perception specific variance was unrelated to age. These models suggest that the age trend identified in Model 1B was essentially due to the negative effects of age on fluid cognitive ability.

In regards to face memory, Model 1B and 2B indicated a strong negative linear trend of age, supporting Hildebrandt et al. (2011) and others who found face memory declines with age. Model 2B indicated that this negative trend remained even when face memory was nested under a general cognitive ability factor. Model 3B indicated that part of the negative relation with age was mediated by the common factor, but specific direct effect of age on face memory remained. Model 3C, however, illustrated that when the common factor was indicated by a stronger fluid cognitive ability factor, that the effects of age could be fully mediated by the common factor and no direct relation of age on nested face memory remained. The common factor estimated in Model 4C differed from the one estimated in Model 3B because the common factor in Model 4C included only the fluid cognitive ability components of immediate and delayed memory and face perception, essentially removing the non-fluid ability aspects of immediate and delayed memory and face perception from the common factor. In Model 4C, the common factor was indicated only by vision, fluid cognitive abilities, physical health, and the nested face memory factor. The remaining face memory variance that was unrelated to vision, general fluid abilities, and physical health did not have an additional negative effect of age. Overall, these results suggest that the age related decline in face memory is fully related to the age related declines in vision, general fluid abilities, and physical health, with no additional effects of age on face memory that are not explained through the common factor.

#### Common Factor and Face Cognition

When ignoring the effects of age or the use of a nested structure for cognitive abilities, we found positive relations between face cognition, fluid cognitive ability, and memory with vision and physical health. However, these relations dropped in magnitude, or were eliminated completely, once we controlled for the linear effects of age. This suggests that in general, some of the relations between face cognition, fluid cognitive ability, and memory, with vision and self-reported physical health are due to age. After controlling for age, the relations between the cognition factors remained as well as the relations between the cognition factors (with the exception of immediate and delayed memory) with vision, suggesting that with increasing age, cognitive factors and vision are still related. However, the only variable still significantly related with physical health was fluid cognitive ability.

Because both, fluid cognitive (or later the general cognitive factor which was marked by fluid cognitive ability indicators) and vision consistently loaded on the common factor, this finding indicates that the common factor established in this work is similar in nature to other models of the common factor (e.g., Christensen et al., 2001). Both face perception and face memory significantly loaded on that common factor indicating that the common variance shared by vision, fluid cognitive ability, and immediate and delayed memory also predicted variance in face perception and face memory. The relations of face cognition with the common factor also remained after controlling for age. This suggests that the common factor includes face cognition.

Of notable importance is how the relations of face perception and face memory with the common factor changed once we controlled for general fluid ability in each construct. Employing a nested model structure, with immediate and delayed memory, face perception, and face memory nested under a general fluid cognitive ability factor, the nested face perception factor was no longer correlated with vision and physical health (Model 2B) or directly related with the common factor (Models 3A–4C). This suggests that the relation of face perception with the common variance of vision, general fluid cognitive abilities, and self-reported physical health is fully explained by general fluid cognitive ability. Face memory, on the other hand, when modeled as a nested factor, was still correlated with vision (Models 2A and 2B) and related to the common factor (Models 3A–4C). These relations held even after controlling for age.

#### Face Cognition and Physical Health

In the final model (Model 4C), the common factor was composed of general fluid cognitive ability, vision, physical health, and nested face memory. The occurrence of the first three variables is typically identified; our model for the first time also shows a loading of a nested face memory factor. The purely correlational models (Models 1A and 1B) showed a relation between face memory with vision and each of the cognitive ability factors, but after controlling for age, face memory was unrelated to physical health. Models 2A and 2B indicates that once the general cognitive ability component of face memory is partialled out, the remaining face memory-specific variance is unrelated to physical health, and Model 4C suggest face memory is only related to physical health through the common factor.

Face perception, on the other hand, is essentially unrelated to self-reported physical health. The first models (Models 1A and 1B) suggest that age fully explains the relation between face perception and physical health. In addition, once we controlled for the general fluid components of face perception, face perception was not significantly correlated with physical health (Model 2B) and was related to the common factor, and thus to physical health (Model 4C). Overall, these results indicate that face perception is unrelated to physical health. Instead, any relation found between the two will most likely be due to either age or to the positive relation between fluid cognitive ability with physical health.

#### Face Cognition and Vision

Our results indicate that face memory is positively related with vision. Model 2B indicates that even after controlling for the general fluid component of face memory, the nested face memory factor is still significantly correlated with vision. Models 1B and 2B indicate that even once age is controlled for, both factors are still positively correlated with one another. The first set of common factor models (Models 3A and 3B) show that face memory is again related to vision through the common factor, even after controlling for age. Finally, the last model (Model 4C) shows that even once the general cognitive ability components of face memory are controlled for, the specific face memory variance is positively related to the common factor and consequently to vision.

The pattern for face perception, on the other hand, is different. Model 1B shows that after controlling for the effects of age, face perception is positively related with vision. However, Model 2A indicates that once we control for the general fluid cognitive ability component of face perception, that the nested face perception factor is unrelated to vision. The first set of common factor models (Models 3A and 3B) show that face perception is positively related to the common factor and thus to vision, again after controlling for the effects of age. However, the last model (Model 4C) shows that only the general fluid aspects of face perception are related to the common factor, and thus to vision, suggesting that the specific face perception variance is unrelated to vision. Thus, the relations found in the Models 1A, 1B, 3A, and 3B is most likely due to age and the general fluid aspect of face perception.

These models suggest that face memory is related to physical health and vision, even after controlling for age and after controlling for general fluid cognitive abilities. Face perception, on the other hand, after controlling for age or for general fluid cognitive ability, is unrelated to physical health and to vision.

### Implications for the Common Cause Hypothesis

Overall, our models illustrate significant shared variance between fluid cognitive abilities, vision, and self-reported physical health. In Models 1A–2B, these constructs were significantly correlated with one another, and in Models 3A–4C, a higher-order factor structure, which was indicated by the fluid cognitive abilities, vision, and self-reported health, had adequate fit to the data. That a single higher-order factor had adequate fit to our data supports findings by Christensen et al. (2001) and others who could model a common factor. However, we also found that controlling for the linear effects of age reduced these relations, supporting findings by Salthouse et al. (1998), and others who found a portion of the shared variance amongst these constructs is attributable to age. We found, like many others, that there is shared variance amongst cognition, vision, and physical health, but that this does not fully explain the relations between these constructs. In other words, a common factor helps explain and model *most* of the shared variance amongst these factors, but not all. For example, Anstey et al. (2001) found that *most* of the age related variance in cognitive ability could be mediated by vision and hearing, except for a weak direct effect (β = -0.27) of age on cognitive ability. Christensen et al. (2001) found that a common factor fully mediated the relation between age and cognition, but *not* between age and vision. We can conclude that a common factor explains most, but not all, of the shared variance, rejecting a strict interpretation of the common cause hypothesis as it applies to social cognition, and instead our results suggests domain general and domain specific aging mechanisms.

### Limitations

While this study has several methodological advantages over other studies (e.g., multiple measures of face cognition and its covariates within the structure of intelligence) there are some limitations. First, this study was based on a cross-sectional sample and did not follow individuals longitudinally. Consequently, a rigorous distinction between age effects and cohort effects was not possible, and could lead to over estimating the effects of age on cognitive abilities. In addition, while we had multiple measures of cognition, we did not include multiple measures of self-reported physical health or vision. A replication of this study that addresses those limitations is needed.

## Conclusion

Research on the common cause hypothesis suggests that we should find age-related declines in fluid cognitive abilities and a relation between fluid cognitive abilities with sensory functions and physical health. However, it is unclear whether this decline should be primarily related to just the fluid ability component of these abilities, or whether it is related to specific constructs themselves, after general fluid cognitive ability was partialed out. This study adds to the literature by examining the relation between vision and physical health with face cognition – which has been established as a specific human ability in previous work. We found that both face perception and face memory significantly loaded on the common factor, thus relating both constructs to physical health and vision. After controlling for the general fluid cognitive ability components of both face cognition variables, we found that the relations of face perception, but not face memory, with vision and physical health could be completely explained by age and by the general fluid cognitive ability components of face perception.

## Conflict of Interest Statement

The authors declare that the research was conducted in the absence of any commercial or financial relationships that could be construed as a potential conflict of interest.

## APPENDIX

**Table A1:** Covariance matrix.

	FM1	FM2	FM3	FM4	REA1	WM1	WM2	FP1	FP2	FP3	FP4	FP5	IDM1	IDM2	IDM3	IDM4	IDM5	IDM6	PF	RP	BP	GH	VA	CON	AGE_C
FM1	0.010																								
FM2	0.010	0.015																							
FM3	0.010	0.012	0.015																						
FM4	0.007	0.008	0.009	0.013																					
REA1	0.036	0.045	0.042	0.037	0.811																				
WM1	0.038	0.043	0.042	0.030	0.557	1.004																			
WM2	0.043	0.054	0.051	0.033	0.562	0.672	0.997																		
FP1	0.006	0.007	0.006	0.005	0.036	0.040	0.043	0.011																	
FP2	0.004	0.004	0.005	0.005	0.024	0.021	0.019	0.004	0.013																
FP3	0.006	0.007	0.007	0.006	0.041	0.043	0.039	0.005	0.005	0.015															
FP4	0.003	0.004	0.004	0.003	0.039	0.039	0.041	0.004	0.003	0.007	0.013														
FP5	0.004	0.004	0.004	0.003	0.028	0.030	0.030	0.003	0.003	0.004	0.003	0.009													
IDM1	0.005	0.006	0.006	0.005	0.054	0.062	0.055	0.004	0.003	0.004	0.003	0.003	0.021												
IDM2	0.008	0.010	0.009	0.008	0.071	0.071	0.070	0.006	0.004	0.006	0.005	0.004	0.017	0.031											
IDM3	0.011	0.013	0.013	0.010	0.091	0.111	0.112	0.008	0.007	0.009	0.008	0.006	0.018	0.021	0.050										
IDM4	0.016	0.019	0.019	0.015	0.134	0.142	0.155	0.013	0.009	0.012	0.011	0.008	0.024	0.031	0.060	0.099									
IDM5	0.010	0.012	0.012	0.009	0.090	0.092	0.105	0.007	0.005	0.009	0.009	0.005	0.015	0.020	0.031	0.048	0.050								
IDM6	0.012	0.015	0.014	0.010	0.111	0.111	0.127	0.009	0.006	0.011	0.011	0.006	0.018	0.024	0.036	0.056	0.050	0.077							
PF	0.011	0.017	0.012	0.008	0.113	0.128	0.136	0.013	0.002	0.010	0.007	0.006	0.012	0.014	0.023	0.039	0.018	0.023	0.227						
RP	0.006	0.008	0.007	0.003	0.046	0.058	0.071	0.005	0.002	0.006	0.003	0.002	0.004	0.006	0.009	0.011	0.003	0.009	0.100	0.126					
BP	0.013	0.014	0.009	0.005	0.112	0.129	0.160	0.010	0.003	0.012	0.011	0.007	0.009	0.012	0.021	0.028	0.013	0.017	0.278	0.224	0.875				
GH	0.022	0.030	0.026	0.018	0.187	0.212	0.192	0.018	0.006	0.015	0.014	0.011	0.016	0.027	0.038	0.051	0.024	0.029	0.193	0.127	0.347	0.671			
VA	0.046	0.052	0.055	0.046	0.367	0.404	0.410	0.041	0.021	0.035	0.022	0.027	0.039	0.058	0.083	0.120	0.063	0.083	0.142	0.038	0.080	0.214	0.998		
CON	0.045	0.049	0.054	0.038	0.302	0.373	0.402	0.036	0.025	0.032	0.023	0.027	0.042	0.058	0.081	0.109	0.079	0.093	0.135	0.045	0.086	0.162	0.656	0.998	
AGE_C	–1.128	–1.479	–1.407	–1.093	–10.500	–10.698	–12.821	–0.982	–0.581	–0.823	–0.568	–0.736	–1.135	–1.690	–2.557	–3.711	–1.900	–2.404	–3.706	–1.395	–3.437	–6.447	–12.714	–11.107	411.302

*FM1, learning and immediate memory of faces; FM2, delayed recognition of learned faces 1; FM3, delayed recognition of learned faces 2; FM4, eyewitness testimony; REA1, Raven’s advanced progressive matrices; WM1, memory updating; WM2, rotation span; FP1, sequential matching of part-whole faces—conditions part; FP2, sequential matching of part-whole faces—conditions whole; FP3, simultaneous matching of spatially manipulated faces—conditions upright; FP4, simultaneous matching of spatially manipulated faces—conditions inverted; FP5, facial resemblance; IDM1, verbal memory—immediate; IDM2, verbal memory – delayed; IDM3, name memory – immediate; IDM4, name memory – delayed; IDM5, address memory – immediate; IDM6, address memory – delayed; PF, physical functioning subscale of the SF12; RP, role physical subscale of the SF12; BP, bodily pain subscale of the SF12; GH, general health subscale of the SF12; VA, visual acuity; CON, contrast sensitivity; AGE_C, age centered.*
